# Outcomes of ST-Elevation Myocardial Infarction in Patients With Opioid Dependence: A National Inpatient Sample Study

**DOI:** 10.7759/cureus.64161

**Published:** 2024-07-09

**Authors:** Abdel-Rhman Mohamed, Ahmad Abdelrahman, Momin Shah, Ayman Salih, Jama Hersi, Abdulmajeed Alharbi, Abdelmoniem Moustafa, Ehab Eltahawy

**Affiliations:** 1 Internal Medicine, The University of Toledo Medical Center, Toledo, USA; 2 Internal Medicine, The University of Toledo College of Medicine and Life Sciences, Toledo, USA; 3 Cardiovascular Medicine, The University of Toledo Medical Center, Toledo, USA

**Keywords:** illicit drug use, national inpatient sample (nis) and the healthcare cost and utilization project (hcup), cad: coronary artery disease, opiate misuse, st-elevation myocardial infarction (stemi)

## Abstract

Background: ST-segment elevation myocardial infarction (STEMI) is a critical condition characterized by the sudden obstruction of one or more coronary arteries, resulting in diminished blood flow to the heart muscle. This acute ischemic event demands swift and precise intervention to minimize myocardial damage and preserve cardiac function. Opioids, a class of potent analgesic medications, are frequently utilized in the management of STEMI-related chest pain. Despite their efficacy in alleviating discomfort, their use in this context warrants careful consideration due to potential adverse effects and interactions.

Methods: In this large nationwide retrospective observational study, we evaluated the effect of opioid dependence on inpatient mortality, length of hospitalization, and cost of hospitalization of patients with STEMI. Data was collected for 2019 from various hospitals across the United States using the National Inpatient Sample (NIS) through the Healthcare Cost and Utilization Project (HCUP). Using the International Classification of Diseases-10 codes (ICD-10), we identified a primary diagnosis of STEMI in patients over the age of 18, as well as a secondary diagnosis of opioid dependence.  Complex samples and multivariable logistic and linear regression models were used to determine the association of opioid dependence on inpatient mortality, length of hospitalization, and cost of hospitalization of patients with STEMI. Of the patients who fit our criteria, we identified other comorbidities and diagnoses associated with them as potential confounders including drug abuse, hypertension, diabetes, alcohol use, obesity, peripheral vascular disease, and chronic lung disease. Other confounders that were adjusted for include race, Charlson Comorbidity index, median household income, insurance, hospital region in the US, hospital bed size, and teaching status of the hospital.

Results: A total of 661,990 patients presented to a hospital with a primary diagnosis of STEMI in 2019. The majority of the patients were male with a mean age of 62.5+/-3.4 and were Caucasian American. Patients who were opioid dependent were found to be on average younger, earned less than the 25th percentile household income, had a higher history of illicit drug and alcohol use, and had Medicaid. They were also found to have higher rates of chronic lung disease at 39.2%, compared to 21.4.% in patients who were not opioid-dependent. Patients who were not opioid dependent were found to have higher rates of hypertension and type 2 diabetes mellitus. Inpatient mortality and cost of hospitalization in STEMI patients with opioid dependence were not statistically different compared to those who were not opioid dependent. However, STEMI patients who were opioid dependent did have an associated longer length of hospitalization.

Conclusion: Opioid use for pain relief in acute coronary syndrome, particularly STEMI, is a mainstay of treatment. Our retrospective cohort dived into assessing the relationship between opioid dependence on its effect on inpatient mortality, length of stay, and cost of hospitalization in STEMI patients. Our study showed that opioid dependence has no significant impact on inpatient mortality. However, it was associated with a longer length of hospital stay in STEMI patients. Further studies may be warranted into the effects of opioid dependence on the length of hospitalization in STEMI patients.

## Introduction

Acute coronary syndromes (ACS), including ST-segment elevation myocardial infarction (STEMI), non-STEMI (NSTEMI), and unstable angina, are responsible for one-third of total deaths in people older than 35 and affect about 15.5 million people in the United States [1]. STEMI is a critical condition characterized by the sudden obstruction of one or more coronary arteries, resulting in diminished blood flow to the heart muscle. This acute ischemic event demands swift and precise intervention to minimize myocardial damage and preserve cardiac function. While the primary treatment for STEMI involves reperfusion strategies such as percutaneous coronary intervention or thrombolytic therapy, adjunctive pharmacotherapy plays a crucial role in managing associated symptoms and optimizing patient outcomes [2].

Opioids, a class of potent analgesic medications, are frequently utilized in the management of STEMI-related chest pain. Despite their efficacy in alleviating discomfort, their use in this context warrants careful consideration due to potential adverse effects and interactions. Opioids exert their analgesic effects primarily through binding to opioid receptors in the central nervous system, thereby modulating pain perception and transmission. By mitigating the intensity of chest pain, opioids offer significant relief to STEMI patients, facilitating their comfort and cooperation during critical phases of diagnosis and treatment. Alternative analgesic strategies, such as nitroglycerin and nonsteroidal anti-inflammatory drugs (NSAIDs), may be considered in select cases to minimize opioid exposure and mitigate potential complications. Bedson et al. investigated the risks associated with long-term opioid prescribing for musculoskeletal pain using data from the UK Clinical Practice Research Datalink. The results revealed dose-dependent increases in serious adverse events including major trauma, intentional overdose, addiction, falls, accidental poisoning, gastrointestinal pathology, and iron deficiency anemia. Higher opioid doses were associated with elevated risks across all outcomes [3].

Despite the efficacy of opioids in alleviating discomfort, their use in ACS warrants careful consideration due to potential adverse effects and interactions. These potential adverse effects and interactions include, but are not limited to, hypotension, CNS depression, and sedation, as well as respiratory drive depression [4]. The administration of opioids in STEMI must be judiciously balanced with several considerations. Firstly, opioids can induce systemic vasodilation and hypotension, which may exacerbate myocardial ischemia and compromise cardiac perfusion [4]. Additionally, these medications can depress respiratory drive and impair oxygenation, particularly in individuals with pre-existing respiratory conditions or compromised pulmonary function. Furthermore, opioids carry a risk of sedation and altered mental status, potentially masking clinical deterioration and delaying timely intervention. Therefore, clinicians must exercise caution when prescribing opioids to STEMI patients, carefully weighing the benefits of pain relief against the risks of adverse effects. This brings to question whether there are adverse outcomes of opioid use, particularly a history of opioid dependence, in the setting of STEMI. What we found was that inpatient mortality and total hospital charges in STEMI patients who were opioid dependent were not statistically different compared to those who were not opioid dependent. However, the length of stay of STEMI patients who were opioid dependent was statistically different compared to those who were not opioid dependent.

## Materials and methods

Study design**

This is a retrospective observational study of patients over the age of 18 hospitalized with a primary diagnosis of STEMI and secondary diagnosis of opioid dependence in various hospitals across the United States in the year 2019.   

Data source and sample 

Data was collected using the National Inpatient Sample (NIS) through the Healthcare Cost and Utilization Project (HCUP). The primary diagnosis was identified using International Classification of Disease-10 codes (ICD). No approval from an ethical committee was warranted as no patient identifiers were used.

Variables of interest 

Of the patients who fit our criteria, we identified other comorbidities and diagnoses associated with them as potential confounders including drug abuse, hypertension, diabetes, alcohol use, obesity, peripheral vascular disease, and chronic lung disease. Other confounders that were adjusted for include race, Charlson Comorbidity index, median household income, insurance, hospital region in the US, hospital bed size, and teaching status of the hospital. 

Statistical analysis

Univariate and multivariable logistic and linear regression models were used to determine the association between opioid dependence on inpatient mortality, length of hospitalization, and cost of hospitalization of patients with STEMI. The statistical analysis was carried out using the STATA version 17.0 software (StataCorp LLC, College Station, Texas, USA). The study was not reported by STROBE guidelines. The data for categorical variables was expressed as percentages, whereas for continuous variables, mean ± SD was used. The test of normality was not performed as it was unable to be done using the STATA software analysis of the National Inpatient Sample. Student's t-test was used for comparing continuous variables, while the chi-square test was used for categorical variables. For the primary and secondary outcomes, unadjusted odds ratios were calculated using univariate regression analysis. To adjust for potential confounders and calculate adjusted odds ratios (aOR), multivariate regression analysis was used. Binary outcomes were analyzed using a logistic regression model, while continuous outcomes were analyzed using linear regression. The models were constructed by incorporating the variables associated with the outcome of interest in the univariable regression analysis, with a cut-off p-value of 0.02. This cut-off was used to be more accurate. All p-values were two-tailed, and the threshold for statistical significance was set at 0.05. 

## Results

Patient demographics**

A total of 661,990 patients presented to a hospital with a primary diagnosis of STEMI in 2019. The majority of the patients were male 61.8% with a mean age of 62.5+/-3.4 and were Caucasian Americans (Table 1). Patients who were opioid dependent were found to be on average younger, earned less than the 25th percentile household income, had a higher history of illicit drug and alcohol use, and had Medicaid as their insurance. They were also found to have higher rates of chronic lung disease at 39.2%, compared to 21.4.% in patients who were not opioid dependent. Patients who were not opioid dependent were found to have higher rates of hypertension and type 2 diabetes mellitus, as well as be on Medicaid (Table 2).

**Table 1:** Demographics of STEMI patients with and without opioid dependence Univariable regression analysis conducted to obtain the p-value. p-value<0.05 indicates statistical significance. STEMI: ST-segment elevation myocardial infarction

Variable	STEMI with Opioid Use	STEMI without Opioid Use	p-value
Number of patients	5428 (0.82%)	656562 (99.18%)	-
Mean age (years)	59.76	66.98	
Female gender (%)	39.26%	37.25%	p=0.1651
Race (%)			p <0.01
Caucasian	72.68%	73.17%	-
Black or African-American	16.65%	11.47%	
Hispanic	7.3%	8.8%	
Asian or Pacific Islander	0.65%	2.9%	
Native American	0.56%	0.62%	
Other	2.15%	3.05%	
Charlson Comorbidity Index (%)			
0	0%	0%	
1	16.41%	22.87%	
2	24.06%	24.34%	
3 or more	59.54%	52.79%	
Median Household Income in patients zip code (%)			p <0.01
0-25th percentile	39.51%	31.09%	
26th-50th percentile	25.83%	26.53%	
51st-75th percentile	23.17%	24.03%	
76th-100th percentile	11.49%	18.35%	
Insurance			p <0.01
Medicare	58.99%	52.66%	
Medicaid	9.56%	27.99%	
Private insurance	26.43%	13.85%	
Self-pay	5.02%	5.5%	

**Table 2:** Comorbidities of STEMI patients with and without opioid dependence. Univariable regression analysis conducted to obtain the p-value. p-value<0.05 indicates statistical significance. STEMI: ST-segment elevation myocardial infarction

Comorbidities	STEMI with Opioid Use	STEMI without Opioid Use	p-value
Drug Abuse (%)	93.92%	2.25%	p<0.01
Hypertension, complicated (%)	47.37%	44.74%	p=0.0955
Hypertension, uncomplicated (%)	33.09%	38.03%	p <0.01
Diabetes, complicated (%)	25.53%	26.93%	p=0.3130
Diabetes, uncomplicated (%)	9.77%	14.33%	p <0.01
Alcohol use (%)	9.68%	3.3%	p <0.01
Obesity (%)	19.63%	20.98%	p=0.2921
Peripheral Vascular Disease (%)	10.69%	10.62%	p=0.9437
Chronic Lung Disease (%)	39.26%	21.35%	p <0.01

Inpatient mortality outcome**

The total number of inhospital mortality was 29630 (4.5%). Inpatient mortality in STEMI patients who were opioid dependent was not statistically different compared to those who were not opioid dependent (aOR 0.798; CI 95% 0.553-1.151, p=0.226). We found that a higher Charlson Comorbidity Index score was related to an increased inpatient mortality (aOR 1.154; CI 95% 1.139-1.169, p=0.01). Other factors that had a significant correlation with increased mortality include age (p=0.01) and Asian and Pacific Islanders (p=0.02), as well as those who self-paid costs of hospitalization (p=0.01) (Table 3).

**Table 3:** Inhospital mortality results of STEMI patients with opioid dependence Univariable regression analysis conducted to obtain the p-value. p-value<0.05 indicates statistical significance. STEMI: ST-segment elevation myocardial infarction

Variable	Adjust Odds Ratio	95% Confidence Interval		p-value
Inhospital mortality with opioid dependence	0.798	0.553	1.151	p=0.226
Age	1.038	1.035	1.042	p <0.01
Race				
Black	0.916	0.833	1.008	p=0.071
Hispanic	0.958	0.869	1.056	p=0.387
Asian or Pacific Islander	1.206	1.031	1.411	p=0.02
Native American	1.151	0.819	1.616	p=0.416
Other race	1.271	1.087	1.487	p=0.003
Median household income in patients zip code (%)				
26th-50th percentile	0.92	0.855	0.991	p=0.027
51st-75th percentile	0.899	0.831	0.974	p=0.009
76th-100th percentile	0.928	0.848	1.016	p=0.106
Charlson Comorbidity Index of 2	1.154	1.139	1.169	P <0.01
Insurance				
Medicaid	1.094	0.962	1.246	p=0.170
Private insurance	0.937	0.855	1.029	p=0.177
Self-pay	1.573	1.341	1.845	p <0.01
Hospital region				
Midwest	0.912	0.829	1.002	p=0.054
South	0.991	0.909	1.081	p=0.837
West	1.119	0.002	0.003	p=0.019

Hospital length of stay 

The average mean of length of stay (LOS) for patients who were not opioid dependent was about 4.39 days, while the average mean of LOS for patients who were opioid dependent was about 5.07 days. The LOS in STEMI patients who were opioid dependent was statistically different compared to those who were not opioid dependent (average mean: 0.617; CI 95% 0.283-0.951, p=0.01). Other factors that also had a significant correlation include age, Asian and Pacific Islanders, a higher Charlson Comorbidity Index, and teaching status of the hospital (p<0.01) (Table 4).

**Table 4:** Inhospital length of stay results of STEMI patients with opioid dependence Univariable regression analysis conducted to obtain the p-value. p-value<0.05 indicates statistical significance. STEMI: ST-segment elevation myocardial infarction

Variable	Coefficient	95% Confidence Interval		p-value
Length of stay in opioid dependence	0.617	0.283	0.951	p<0.0
Age	0.009	0.008	0.012	p<0.0
Female gender	-0.054	-0.112	0.005	p=0.072
Race				
Black	0.049	-0.073	0.172	p=0.426
Hispanic	0.131	-0.012	0.274	p=0.073
Asian or Pacific Islander	0.468	0.235	0.701	p<0.0
Native American	0.177	-0.188	0.542	p=0.341
Other	0.452	0.198	0.706	p <0.01
Median household income in patients zip code (%)				
26th-50th percentile	-0.049	-0.141	0.043	p=0.295
51st-75th percentile	-0.094	-0.199	0.011	p=0.078
76th-100th percentile	-0.139	-0.259	-0.02	p=0.022
Charlson Comorbidity Index of 2	0.609	0.586	0.633	p <0.01
Teaching institution	0.859	0.776	0.942	p <0.01
Hospital region				
Midwest	-0.52	-0.718	-0.322	p <0.01
South	-0.148	-0.341	0.046	p=0.135
West	-0.741	-0.947	-0.535	p <0.01
Hospital size				
Medium bed size	0.463	0.338	0.587	p <0.01
Large bed size	1.25	1.11	1.39	p <0.01

Hospital cost 

Opioid dependence was not associated with a significant increase in total hospital charges when compared with those who were non-opioid dependent (average mean: -7306.439, CI 95% -14897.28-284.406, p=0.059). Factors that were statistically significant that affected the cost of hospitalization include age, female gender, a higher Charlson Comorbidity Index, and teaching status of the hospital (p<0.01) (Table 5).

**Table 5:** Hospital admission total charges results of STEMI patients with opioid dependence Univariable regression analysis conducted to obtain the p-value. p-value<0.05 indicates statistical significance. STEMI: ST-segment elevation myocardial infarction

Variable	Coefficient	95% Confidence Interval		p-value
Total charges in opioid dependence	-7306.439	-14897.28	284.406	p=0.059
Age	-482.611	-548.006	-417.216	p <0.01
Female gender	-13782.38	-15326.48	-12238.28	p <0.01
Race				
Black	-9761.547	-13945.7	-5577.398	p <0.01
Hispanic	15563.39	9098.454	22028.32	p <0.01
Asian or Pacific Islander	20141.11	9498.198	30784.02	p <0.01
Native American	-3460.156	-16580.18	9299.869	p=0.581
Other	24368.07	14322.09	34414.05	p <0.01
Median household income in patients zip code (%)				
26th-50th percentile	1016.861	-2522.588	4556.31	p=0.573
51st-75th percentile	2089.921	-2147.656	6327.49	p=0.334
76th-100th percentile	6278.453	458.372	12098.53	p=0.034
Charlson Comorbidity Index of 2	6845.82	6192.352	7499.304	p <0.01
Teaching institution	21304.45	17747.99	24860.92	p <0.01
Hospital region				
Midwest	-11779.65	-19978.52	-3580.789	p=0.005
South	11287.83	2528.583	20047.08	p=0.012
West	27953.51	16593.46	39313.56	p <0.01
Hospital size				
Medium bed size	13848.11	7202.13	20494.08	p <0.01
Large bed size	29611.93	23031.66	36192.20	p <0.01

## Discussion

Our study looks at the outcomes of patients admitted for STEMI in those who are opioid naïve compared to those with a history of opioid use. What we found was that inhospital mortality was not significantly different in patients with opioid dependence as compared to patients without. However, opioid dependence was associated with a statistically significant longer length of hospital stay.

In the context of ever-increasing opioid dependence and cardiovascular diseases, including STEMI in the US, this study provides new information and understanding of what effects these two conditions have on hospital stay, mortality, and cost. Pain management in the acute phase of ACS is recommended according to European and American guidelines [5,6]. Morphine and other opioids have widely been used in daily practice in STEMI patients. In our study, we found an increased length of hospital stay for opioid-dependent patients compared to non-opioid-dependent patients. This is consistent with previous studies that have shown poor prognosis after STEMI in patients with opioid dependence. Our study showed that opioid dependence is associated with an increased LOS, which sheds some light on possible opioid-related side effects on STEMI patients. To the best of our knowledge, this is the largest analysis to date that explores the outcomes of STEMI in this patient population. This study considered demographic factors (age, race, gender, insurance type, household income), the severity of illness, inhospital mortality, total hospital charges, and LOS in both opioid dependents and nonopioid dependents. Trends of coexisting comorbidities among STEMI hospitalizations were also explored. Recent evidence suggests that opioids may delay and inhibit the absorption of antiplatelet agents [7-9], which may have a more pronounced impact on clinical outcomes in STEMI patients.

While the exact mechanism is not well understood, there have been a few theories posed as to why opioid use may worsen outcomes in those with ST-elevation myocardial infarctions. Those who present with ST-elevation myocardial infarctions are typically patients with suboptimal cardiovascular health, to begin with, and so several may have previous stents placed or are taking platelet inhibitors. It is known that coronary stent re-thrombosis infarctions are associated with increased mortality [4,10].

A cohort study by Korsgaard et al. found that patient outcomes are worse for opioid-dependent patients compared to non-opioid users [11]. Although there are several studies showing worse STEMI outcomes for opioid users, there are no randomized clinical trials or any robust studies that show a clear association between poor STEMI outcomes and chronic opioid use. In other studies, opium consumption was associated with the increased odds of both ischemic heart disease and myocardial infarction (MI) diseases and mortality. These findings were consistent with similar studies. Niaki et al. showed that opium consumption was a significant risk factor for MI with an AOR of 26.3 [12]. Sadeghian et al. presented that opium abuse was a major risk factor for ischemic heart disease [13].

The 2018 review article by Tavenier et al. found that morphine’s effects on the GI tract inhibit the absorption of orally administered platelet inhibitors which are vital for treatment [14]. Patients with right-sided STEMI and those involving the right coronary artery with right ventricular failure are pre-load dependent and can have fatal outcomes when morphine, a known vasodilator, is given as it accentuates these hemodynamic changes. Those patients on chronic opioids have an increased risk of respiratory depression because respiratory depression tolerance is slower and more prolonged than analgesic tolerance seen in this subset of patients. This risk of respiratory depression will in turn put the patient at higher risk for hypoxemia, which is not prognostically favorable in STEMI patients. The heart has opioid receptors, and endogenous opioid peptide (EOP) dynorphin has also been found in the heart. These EOPs are released during myocardial ischemia and can cause arrhythmias, bradycardia, and hypotension. In a 1993 journal article from the European Heart Journal, it was concluded that opioid agonists potentiated the fatal complications secondary to myocardial ischemia, giving yet another possible mechanism for the worse outcomes reported [4]. Furthermore, a 2013 Journal of Internal Medicine article conducted a nested case-control study to answer the question of whether opioid use for non-cancer patients was associated with an increased risk for MI among adults [10]. The primary working theory from that study was whether the risk of MI was related to opioid-induced hypogonadism. The thinking is that since the morbidity and mortality rates from CAD are 2.5 to 4.5-fold higher in men than in women, with the gap narrowing after menopause, there must be some level of influence that sex hormones have on the cardiovascular system. They reported that the use of opioid prescriptions was associated with a 1.53-fold risk of MI amongst men, whereas no association was seen amongst women. They found a 1.3-fold increase in MI for current opioid users overall.

In our study, there were no significant differences in inhospital mortality rates between opioid-dependent and non-opioid-dependent patients. However, higher Charlson Comorbidity Index scores, Asian and Pacific Islanders, and out-of-pocket payment for hospitalization were associated with increased inpatient mortality. The bulk of these patients were young white males with a history of polysubstance use and comorbid lung conditions. In contrast to our findings, Ranka et al. found higher all-cause mortality among patients who were opioid-dependent compared to non-opioid-dependent patients [15]. In line with this, a cohort study to assess the one-year mortality of opioid users who had myocardial infarction Korsgaard et al. demonstrated that the high frequency of comorbid conditions in opioid-dependent patients contributes to their all-cause mortality [11]. More studies are needed to better elucidate the mortality rates of opioid-dependent patients with STEMI.

The combined cost of cardiovascular disease and stroke was $327 billion between 2013 and 2014 [16]. In addition, the economic cost of opioid use disorder was $1.02 trillion [17]. Our study found no significant difference in total charges in STEMI patients who were opioid dependent compared to those without opioid dependence.

Now, while there is a scarcity of information on the actual pathophysiology, it resoundingly seems that there are multiple factors contributing to the reported worse outcomes in STEMI patients with opioid use as opposed to those without. With the ubiquity of long-term opioid usage, and the ever-growing number of acute coronary syndrome cases, the interplay and prognostic implication one has on the other is a medical curiosity needing to be pondered. In the literature, opioid use has been shown to increase the risk for MI by nearly 2.25-fold compared to those using NSAIDs. With such figures, it is important to continue research on this topic and fill the paucity of information regarding it.

Limitations 

Given its observational nature, there is a possibility for selection and unmeasured bias. However, this was controlled using a p-value of less than 0.05. Additionally, information on out-of-hospital mortality, short-term outcomes that did not lead to hospitalizations, and long-term outcomes were not available in the NIS dataset. Despite these limitations, the NIS database provides an invaluable population-based resource for evaluating national trends.  

## Conclusions

Opioid use is one of the pillars for pain relief in ACS, particularly STEMI. Our retrospective cohort dived into assessing the relationship between opioid dependence on its effect on inpatient mortality, LOS, and cost of hospitalization in STEMI patients. Our study showed that opioid dependence has no significant impact on inpatient mortality. However, it was associated with a longer length of hospitalization and in patients with STEMI. Further studies may be warranted into the effects of opioid dependence on the length of hospitalization in STEMI patients.
